# Band Gap Measurements of Nano-Meter Sized Rutile Thin Films

**DOI:** 10.3390/nano10122379

**Published:** 2020-11-29

**Authors:** Nikolaos C. Diamantopoulos, Alexandros Barnasas, Christos. S. Garoufalis, Dimitrios I. Anyfantis, Nikolaos Bouropoulos, Panagiotis Poulopoulos, Sotirios Baskoutas

**Affiliations:** 1 Materials Science Department, University of Patras, 26504 Patras, Greece; nikosdiamadopoylos@gmail.com (N.C.D.); mparnalex@gmail.com (A.B.); garoufal@upatras.gr (C.S.G.); up1057157@upatras.gr (D.I.A.); nbouro@upatras.gr (N.B.); 2 Foundation for Research and Technology Hellas, Institute of Chemical Engineering and High Temperature Chemical Processes, 26504 Patras, Greece; 3 Institut für Physikalische Chemie, Universität Hamburg, Grindelallee 117, 20146 Hamburg, Germany

**Keywords:** thin films, rutile, semiconductor oxides, optical properties, quantum confinement, potential morphing method

## Abstract

Thin Titanium films were fabricated on quartz substrates by radio frequency magnetron sputtering under high vacuum. Subsequent annealing at temperatures of 600 ${}^{\circ }$C in air resulted in single-phase ${TiO}_{2}$ with the structure of rutile, as X-ray diffraction experiment demonstrates. Atomic-force microscopy images verify the high crystalline quality and allow us to determine the grain size even for ultrathin ${TiO}_{2}$ films. Rutile has a direct energy band gap at about 3.0–3.2 eV; however, the transitions between the valence and conduction band are dipole forbidden. Just a few meV above that, there is an indirect band gap. The first intense absorption peak appears at about 4 eV. Tauc plots for the position of the indirect band gap show a “blue shift” with decreasing film thickness. Moreover, we find a similar shift for the position of the first absorbance peak studied by the derivative method. The results indicate the presence of quantum confinement effects. This conclusion is supported by theoretical calculations based on a combination of the effective mass theory and the Hartree Fock approximation.

## 1. Introduction

Titanium dioxide $TiO_{2}$ shows a wide range of properties for both basic and applied research. It has been widely studied for photocatalytic water splitting, solar hydrogen generation, lithium-ion batteries, dye-sensitized solar cells, photovoltaics, supercapacitors, self-cleaning nano-paints, etc., offering a wide range of possible technological and life improving applications. A similarly large field of applications is also possible for $TiO_{2}$ nanoparticles due to their high performance properties in water and air purification, toothpaste, antibacterial protection, decontamination, photocatalysis, sensing and paints [1]. $TiO_{2}$-based photocatalysts are among the most studied materials for photocatalytic applications due to their interesting electronic properties, eco-friendliness, stability and cost-effectiveness [2,3]. Accordingly, many efforts have been focused on synthesizing new $TiO_{2}$-based materials for various applications [4,5,6,7,8]. Moreover the reduction of size and dimensionality of the fabricated samples introduces quantum effects such as tunneling and information confinement and properties which depend on the quantum description of chemical bonding at nano-level [9,10].

Extensive research has been conducted through the past years about the crystalline structure of rutile and its dielectric constant. Moreover, a large number of investigations with regard to its semi conductivity, magnetic and optical behavior of reduced and “doped” specimens [11] has been performed. A substantial survey of the literature on rutile up until 1958 has been made by Grant [12]. A good part of this article refers to the defect structure and electronic properties of reduced $TiO_{2}$. Rutile is a dipole-forbidden direct gap semiconductor with a band gap of about 3.0–3.2 eV. That is, the transition probability from the top of the valence band to the bottom of the conduction band is very small [11]. Therefore, the optical absorption at the band gap is negligible. However, its energy is very close to the value of the indirect allowed band gap [13,14]. Due to the weak strength of the direct forbidden transition, the indirect allowed transition dominates in the optical absorption just above the absorption edge. As a result of these intricacies, there are only a few works in literature reporting on the determination of the indirect band gap of rutile in thin film, nanocrystalline or nanoparticle form by optical measurements and relevant Tauc plots [15], see, for example, Refs. [14,16,17,18,19,20,21,22] earlier optical measurements of Cardonna and Harbeke on high quality single crystals showed an intense increase in the absorbance at energies after about 3.7 eV [23]. Those were interpreted in the framework of ab initio theoretical calculations of Glassford and Chelikowski [24].

In this work we discuss the band gap and quantum confinement effects on ultrathin rutile $TiO_{2}$ films fabricated by annealing of titanium films at 600 ${}^{\circ }$C. We determine the absorption edge (indirect band gap) with the help of Tauc plots. We find an increase of the band gap position (“blue shift”) as the film thickness decreases. Moreover, we record intense absorption peaks at energies after about 3.7 eV. For the thickest ultrathin film of 15 nm the UV-visible optical absorption spectrum shows an abrupt increase and consists of at least three Gaussians. The energy point of maximum rate in the increase of the absorption of the first Gaussian is determined via the derivative method. It also shows a “blue shift” with decreasing film thickness. These “blue shifts” are interpreted as evidence for quantum confinement effects.

The aforementioned conclusion is corroborated by theoretical calculations in which the electron and the hole of the system are treated by the Hartree Fock (HF) approximation [25,26,27,28,29,30,31,32]. The resulting HF equations incorporate the effective masses of the two particles and they are numerically solved by he potential morphing method (PMM) [29,30,31,32]. Prior experience has revealed that the adopted methodology has an excellent performance in capturing (quantitatively) the quantum confinement effects.

## 2. Materials and Methods

### 2.1. Experimental Details

Ultrathin $TiO_{2}$ films with thickness t ranging between of 0.4–15 nm were fabricated after 30 min annealing of metallic Ti films at 600 ${}^{\circ }$C in air. A muffle furnace was used for the annealing (model Linn elektronik VMK 22). One thick film (t = 400 nm) was prepared after two hours annealing; it served as a prototype for X-ray diffraction (XRD) characterization. This method of preparation of metal oxides after high temperature oxidation of metals in a furnace in air is well-distributed in literature see, for example, Refs. [28,29,30,31]. Another thick film (t = 115 nm) was used as reference for the determination of the optical band gap of the bulk rutile. The Ti films were deposited on quartz glass by radio frequency (r.f.) magnetron sputtering. The Ti-foil (thickness 0.032 mm) sputtering target (Alfa Aesar, 2-inch, 99.7% purity) was placed on the Torus 2 HV sputtering source of Kurt J. Lesker Company. The deposition temperature was the room temperature. The best pressure could be achieved, with the help of a mechanical and a turbomolecular pump, in our vacuum chamber was $3\times 10^{-5}$ Pa. The Ar partial pressure during deposition was kept at 0.2 Pa. Thin film morphology was monitored with the help of Atomic Force Microscopy (AFM) images. Our AFM is a multimode microscope controlled by Nanoscope IIIa. It is equipped with a 120 μm × 120 μm magnet-free scanner (model AS-130VMF) developed by Bruker (Santa Barbara, CA, USA). The microscope was handled in the non-contact mode [33].

The film thickness was determined by measuring the profile of a narrow scratch [29,34]. After this, we could determine film thickness with an accuracy of about ±5%. With this information, a quartz balance system (Inficon XTM/2) could be calibrated and used for measuring film thickness with an accuracy of ±0.1 nm. Structural characterization of $TiO_{2}$ was carried out via XRD with the help of Bruker, D8-Advance standard powder diffractometer. This was providing Co- and Ni-filtered $CuKa_{1}$ radiation (λ = 0.154059 nm). The specimens were scanned at a step of 0.02${}^{\circ }$ and a scan speed of 10 s/step. The ultraviolet spectra were probed at room temperature (R.T.) in the transmission geometry via a Shimadzu UV-Vis Spectrophotometer. This was the Model: UV 1800 (Shimadzu, Kyoto, Japan) at wavelengths 200–1100 nm.

### 2.2. Theoretical Calculations

The two particle system consisting of one electron and one hole is described by the Hamiltonian

(1) $$-\frac{\hbar^{2}}{2m_{e}^{*}}\nabla_{e}^{2}-\frac{\hbar^{2}}{2m_{h}^{*}}\nabla_{h}^{2}+V_{0}^{e}\left(r_{e}\right)+V_{0}^{h}\left(r_{h}\right)-\frac{e^{2}}{\epsilon }\frac{1}{r_{eh}}$$

$m_{e}^{*}$ and $m_{h}^{*}$ being the electron and hole effective masses respectively. The dependence of the dielectric constant on the dimensions of the system is incorporated in the ϵ parameter [27], while $V_{0}^{\left(h,e\right)}\left(r_{\left(h,e\right)}\right)$ are the confining potentials of the hole and the electron respectively. Besides the confining potential the term U of the resulting HF equations

(2) $$\left[\frac{p_{\left(e,h\right)}^{2}}{2m_{\left(e,h\right)}^{*}}+U_{\left(e,h\right)}\left(r_{\left(e,h\right)}\right)\right]\Phi_{\left(e,h\right)}\left(r_{\left(e,h\right)}\right)=E_{\left(e,h\right)}\Phi_{\left(e,h\right)}\left(r_{\left(e,h\right)}\right)$$

includes both the the exchange and the Coulomb interactions between the two particles. After the numerical solution of the above equations, the exciton total energy is calculated by the expression $E\left(X\right)=E_{h}+E_{e}$ and the material’s effective gap is determined by the relation $E_{g}^{eff}=E_{g}^{bulk}+E\left(X\right)$. The details of the calculations and solution methods are presented elsewhere [32].

## 3. Results

**Figure 1:**
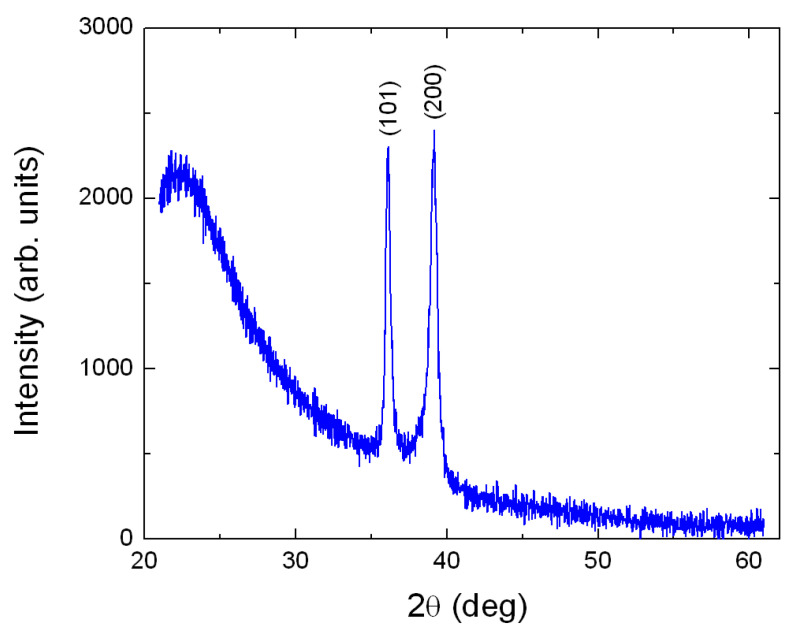
X-ray diffraction pattern of a Ti film grown on quartz glass after annealing for two hours at 600 ${}^{\circ }$C in a furnace under air atmosphere. Pure rutile 400 nm thick was formed. All XRD peaks are identified with the help of JCPDS Card No. 21-1276 (Joint Committee on Powder Diffraction Standards, International Center for Diffraction Data) for pure polycrystalline rutile.

**Figure 2:**
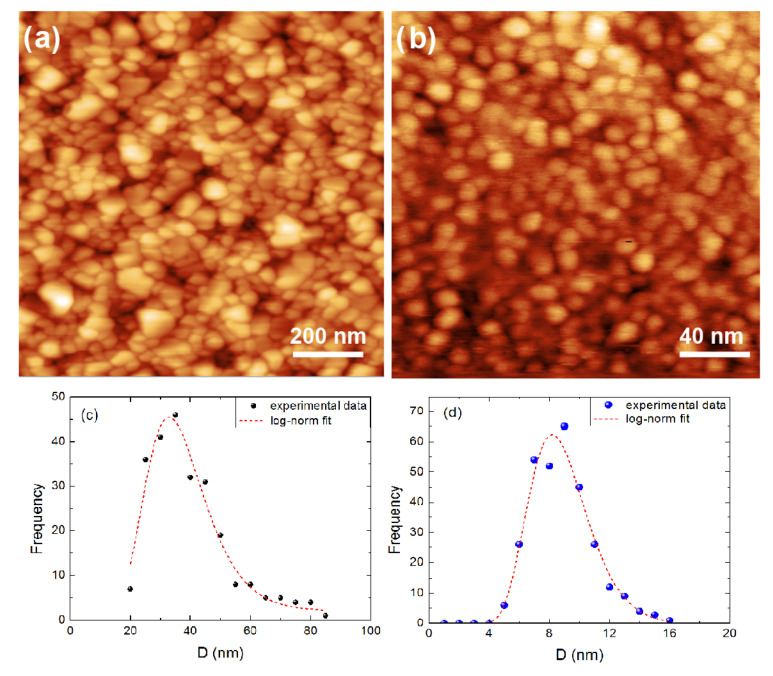
Atomic Force images of (**a**) 400 nm and (**b**) 4.7 nm thick rutile films. In (**c**,**d**) the corresponding experimental-data distributions of the grain size and the log-normal fittings are provided.

An XRD experiment was performed on a 400 nm thick $TiO_{2}$ film. All diffraction peaks of the XRD pattern of Figure 1 were identified with the help of a reference bulk polycrystalline rutile film (JCPDS Card No. 21-1276 (Joint Committee on Powder Diffraction Standards, International Center for Diffraction Data)). Therefore, our samples are single-phase rutile. Both films are polycrystalline; nanocrystallites were monitored on the film surface via AFM experiments for the aforementioned thickest film, Figure 2a as well as for a 4.7 nm ultrathin rutile film, Figure 2b. The grains seem to be quite homogeneous in terms of size. Indeed, this may be observed as it follows: In Figure 2c,d it is exhibited the grain-diameter D size-distribution of the two films of Figure 2a,b, respectively. The log-norm function fits well the experimental data [35]. The average value of D is 33 nm and 8.2 nm for the 450 and 4.7 nm thick films, respectively. The distributions are narrow with respect to magnitude of the crystallite diameter; indeed, the full-width at half-maximum (FWHM) is 25 nm for the first film and 4.5 nm for the second. The root-mean-square roughness (Rrms) for the 4.7 nm thick film is remarkably small, only 0.5 nm, which is a typical value for very thin continuous films [36].

**Figure 3:**
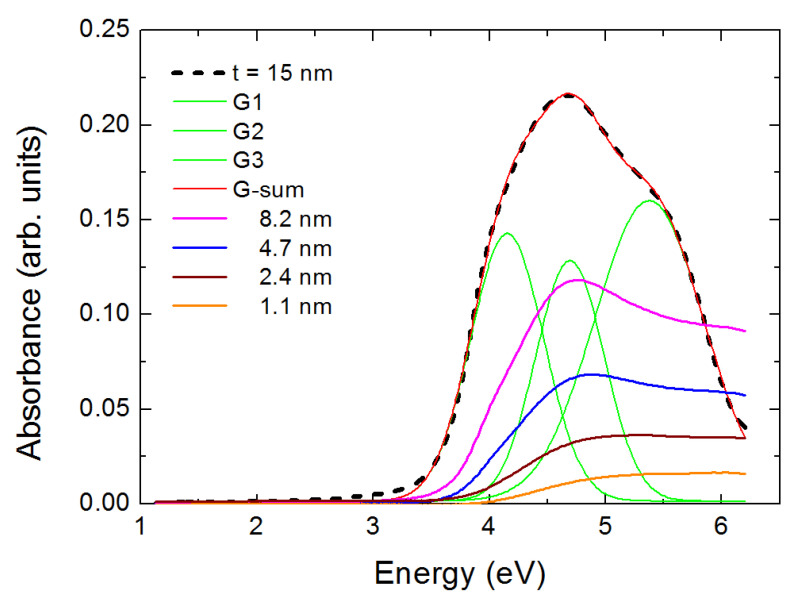
Absorbance spectra for five rutile films; their thickness t is indicated. The absorbance of the thickest film is fitted with the help of three Gaussians G1, G2, G3. As t decreases the spectra present a “blue shift”.

In Figure 3 we show the optical density (absorbance A = −logT, T is the transmittance) spectra [15], for five rutile films with thickness t as indicated. For the 15 nm film one may observe that its spectrum is practically located between about 3 and 6.2 eV (upper limit of our measurement). It is straight forward that this spectrum may be fitted by a minimum set of three Gaussians G1, G2 and G3. After the fitting process one may test that the sum of the three Gaussians coincides with the experimental spectrum. The peak of them is located at 4.15, 4.68 and 5.37 eV, respectively. Room temperature measurements with polarized light determined the imaginary part of the dielectric constant of single-crystalline rutile along and across the c-axis of its tetragonal structure [23]. Those measurements were discussed with respect to ab initio theoretical calculations in [24]. Direct comparison of these measurements to ours show that, within the experimental accuracy, our first Gaussian coincides with the features A (electric field of light parallel to the c axis) and A1 (electric field of light perpendicular to the c axis of the tetragonal rutile system) of [23,24]. On the other hand, the third Gaussian in our spectrum coincides with the feature A2 [23,24]. Between A1 and A2 theory predicts the existence of two more peaks. These were not observed in [23]. Our G2 coincides with the first of these two theoretical predicted features and corresponds to transitions between valence bands 14–15 to the first two conduction bands along the Σ direction near Γ.

**Figure 4:**
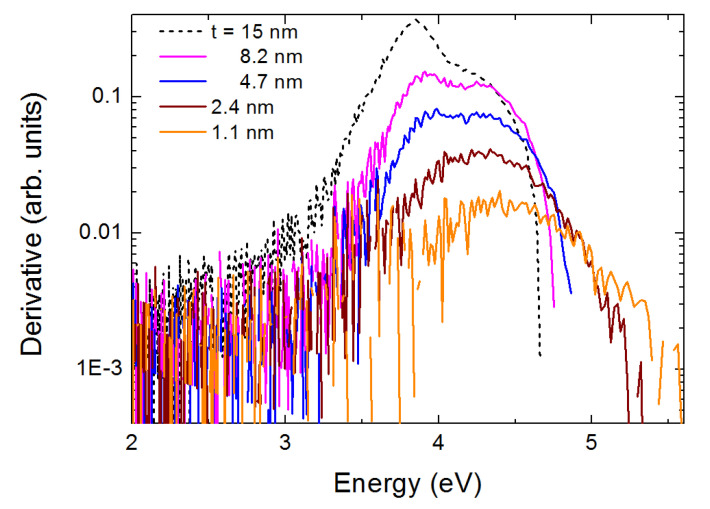
First derivative of the absorbance of the five films of Figure 3; their thickness t is indicated. A logarithmic scale is selected for the Y-axis for better presentation. The maximum of the derivative shows a “blue shift” with decreasing film thickness.

**Figure 5:**
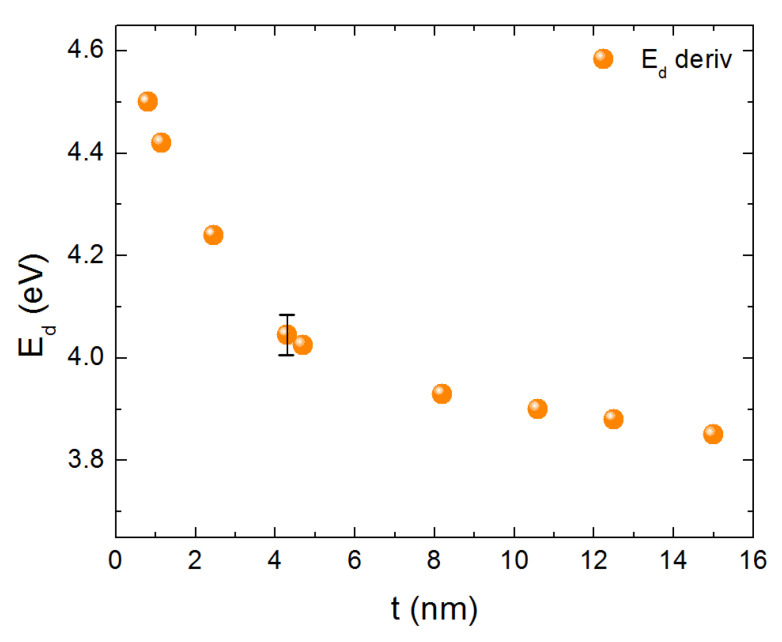
Position of $E_{d}$ as a function of energy. The data reveal a “blue shift”.

In Figure 4 one may observe a “blue shift” of the spectra as the film thickness decreases. This may be viewed more easily if one plots the position Ed of the first derivative of the absorbance of the spectra, appearing in Figure 4 in a logarithmic scale (for sake of clarity of presentation). Ed actually represents the maximum absorbance increase rate of the first peak of each spectrum. The maximum of the derivative shows a “blue shift” with decreasing t. This is presented in Figure 5 and may be interpreted as an evidence for quantum confinement effects.

**Figure 6:**
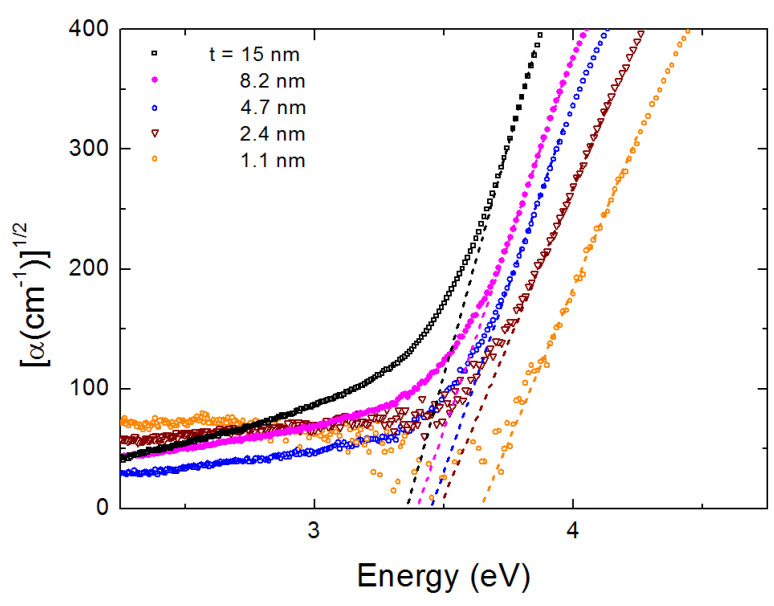
Tauc plots (${\left(\alpha \right)}^{1/2}$ as a function of E) for five rutile films; their thickness t is indicated. A “blue” shift of Eg determined by the Tauc plots is observed.

**Figure 7:**
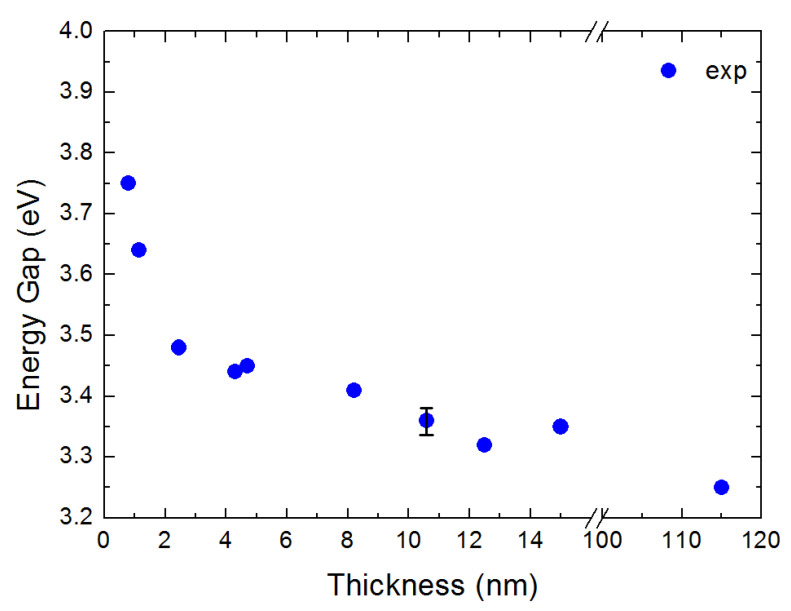
Position of $E_{g}$ as a function of energy. The data reveal a “blue shift”.

As a next step, in Figure 6 we provide Tauc plots for the determination of the position of the indirect band gap $E_{g}$ of our rutile films. These are plots of the ${\left(\alpha \right)}^{1/2}$ as a function of energy E for the five films of Figure 3; α is the absorption coefficient. The intercept of the linear part of the plot with the energy axis determines the position of $E_{g}$. Finally, in Figure 7 we show the results of the Tauc plots for $E_{g}$. One may clearly see an increase in the position of the indirect band gap with the decrease of t. This may be observed if one moves towards the thinner film limit, where quantum confinement effects may be of importance. This is due to the fact that the film thickness approaches the Bohr diameter of the excitons. In the following session comparison between experiment and theoretical calculations is attempted for further physical insight of the indirect band gap of rutile.

On the theoretical part, the calculations were performed adopting the following set of parameters: $m_{e}=3m_{0}$ [13], $m_{h}=0.8m_{0}$ [37] while for the dielectric function the size dependence was captured by adopting the Hanken model [38] with the parameters $\epsilon_{0}=114$ [39], $\epsilon_{\infty }=12$ [37] and $\omega_{LO}=45.34$ meV [40].

**Figure 8:**
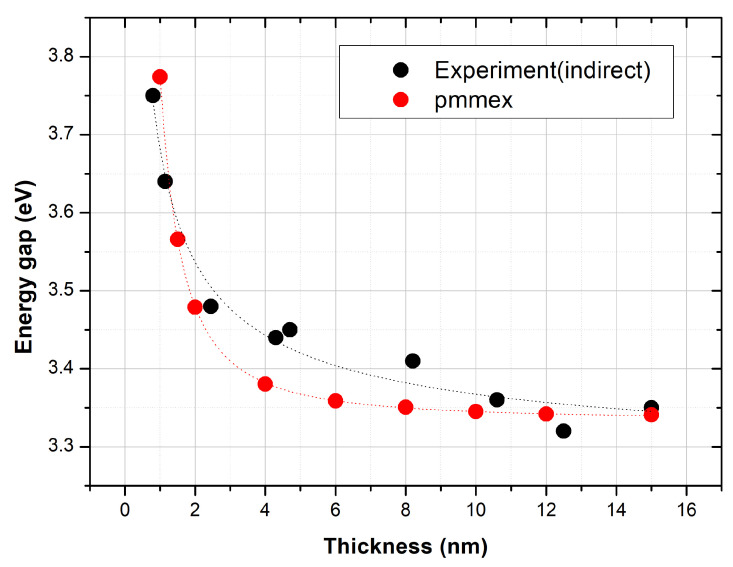
Variation of the theoretically predicted gap as a function of sample thickness t along with experimental data for the indirect gap.

The variation of the gap with regard to the film thickness is presented in Figure 8. For sake of comparison, the experimental data for the indirect gap are also presented in the same graph.The two curves agree well with each other exhibiting similar behaviour with previous studies in confined systems [26,27,28,29,30,31,32]. This behavior strongly suggests that the shifts observed experimentally are evidence of quantum confinement.

## 4. Conclusions

Quantum confinement effects in rutile ultrathin films were studied by experiment and the HF/EMA/PMM combination of theoretical approximations. The rutile thin films have been prepared by oxidizing Ti ultrathin films at 600 ${}^{\circ }$C in air. both the indirect allowed band gap at ∼3.2 eV, and the optical peak at about 4 eV show “blue shifts” with decreasing film thickness. This tuning of optical properties with film thickness is a desired property of nanoscaled semiconductors for being implemented in nanodevices and various applications.
